# Experimental Demonstration of the Use of Peptomangan—Gude

**Published:** 1900-06

**Authors:** 


### Abstracts.

### EXPERIMENTAL DEMONSTRATION OF THE USE OF PEPTOMANGAN—GUDE.

WILLIAM KRAUSS, of Memphis, Tenn. (Memphis *Med. Monthly*), recently made experimental tests of various iron preparations, officinal and nonofficinal, before the Memphis Medical Society. The following account of the tests is of interest to the clinician. He says:

Beginning with the organic double salts, of which the scale salts are representatives, we notice upon the addition of this gastric juice, that a precipitate is formed; the double salt is decomposed and ferric salt remains, which is insoluble, both in gastric and intestinal juice.

The tincture of ferric chloride will precipitate some of the gastric constituents, though most of the iron will remain in solution in the hydrochloric acid; the iron still in solution will not be absorbed, because its nondiffusibiltity is taken advantage of in the manufacture of dialised iron, the acid passing through the animal membrane; when the iron finally reaches the intestine, the alkaline carbonates promptly precipitate it. Ferrous sulphate behaves similarly. In both instances, as you see, the very insoluble ferric oxides finally formed. If you have ever tried to remove iron stains from your water pitcher, you have some idea how insoluble it is.

The insoluble compounds, like reduced iron, or Vallets' mass, only serve to render inert the arsenic with which they are usually prescribed; if dissolved at all in the stomach, they are reprecipitated in the intestine.

Taking now Gude's preparation, we find it soluble, not only in all these reagents, but also in a mixture of them. Potassium ferrocyanide readily gives the iron reaction, excess of ammonia will separate it, redissolving the manganese, which is then recognised by the color of its sulphide, the alkaline copper solution gives the reaction for pepton, showing that it is what the label says. It mixes with arsenious acid, forming a perfect solution, thus giving us a most useful hematopoietic agent. The soluble alkaloids are perfectly soluble in it, as is also mercuric chloride. Being a pepton, it is readily diffusible by osmosis.

The only disturbing agent in the intestinal tract is hydrogen sulphide; this will precipitate it, but, presumably, much of the iron must have been absorbed before it encounters the gas; if not, appropriate agents should be used for its elimination.

Therapeutically, it does not nauseate, constipate, discolor the teeth, precipitate the digestive agents, nor become inert from contact with them. As to the clinical results, I need not add anything to the many reports already on record.

